# Changes in salivary oxidative status, salivary cortisol, and clinical symptoms in female patients with temporomandibular disorders during occlusal splint therapy: a 3-month follow up

**DOI:** 10.1186/s12903-019-0791-8

**Published:** 2019-06-06

**Authors:** E. Vrbanović, I. Lapić, D. Rogić, I. Z. Alajbeg

**Affiliations:** 1 Department of Removable Prosthodontics, School of Dental Medicine, University of Zagreb, Gunduliceva 5, HR-10000 Zagreb, Croatia; 2 Department of Laboratory Diagnostics, University Hospital Center Zagreb, Zagreb, HR-10000 Croatia

**Keywords:** Temporomandibular disorders, Oxidative stress, Salivary cortisol, Occlusal splint, Salivary diagnostics

## Abstract

**Background:**

Differences in the expression of oxidative stress (OS) markers between female patients with temporomandibular disorders (TMD) and healthy individuals indicate that OS plays a role in the pathogenesis of TMD. Because chronic exposure to stress generates oxidative damage during continuous stimulation of the hypothalamic-pituitary-adrenal axis, we expected that higher levels of cortisol might be associated with higher oxidative damage. Our aim was to test the association between OS markers, stress perception, and salivary cortisol (SC) in chronic, female TMD patients. We tracked changes in OS markers and SC during occlusal splint therapy in order to evaluate the influence of treatment on oxidative status. We hypothesized that the effects of TMD therapy would differ among individuals depending on the source and intensity of pain.

**Methods:**

Sixteen female patients were recruited, and 12 finished the study. Clinical assessment and saliva sampling were performed at the baseline and follow-up appointments. Repeated measures analysis of variance and Pearson’s correlation were used for analyzing the data.

**Results:**

After 3 months, a significant reduction in afternoon total antioxidant capacity (TAC) was observed (*p* < 0.05). A significant reduction in afternoon malondialdehyde (MDA) (*p* = 0.021) and a decrease in afternoon MDA to superoxide dismutase ratios (*p* = 0.017) were present in high-intensity pain patients. At baseline, higher levels of perceived stress were significantly associated with higher morning cortisol (*ρ* = 0.67). At the end of the therapy,  reduced  perceived stress was positively correlated with morning SC changes when considering all TMD patients, but the association between perceived stress with OS markers was present only in myofascial pain (MP) group. The effect of treatment on the self-perceived quality of life was more pronounced in female MP patients while the reduction of spontaneous pain was significantly greater in high-intensity pain patients.

**Conclusion:**

Our data indicate that occlusal splint therapy in female TMD patients contributes to increasing their capacity to remove free radicals. The question remains whether or not TAC decreases in this process as a result of avoiding unnecessary processes, once the increase in antioxidants effectively compensates for OS. The intensity and the source of pain should be considered important factors in future investigations evaluating salivary OS markers and their association with perceived stress and SC in TMD patients.

**Trial registration:**

ClinicalTrials.gov NCT03029494. Registered on 2017-01-19.

## Background

Temporomandibular disorders (TMD) encompass the most common painful musculoskeletal and neuromuscular orofacial conditions and affect jaw joints, masticatory muscles, and the surrounding structures. These conditions are still primarily diagnosed through medical history and clinical examination due to their multifactorial pathophysiology, which is still not completely clear [1]. Symptoms, signs, and behavioral risk factors are examined using well-defined, evidence-based questionnaires and protocols that allow for few diagnostic errors and are therefore considered valid methods for TMD diagnosis [2]. The majority of TMD patients are women, with the prevalence rate being the highest between 20 and 40 years of age [3]. The most common diagnoses representing pain-related TMD are myofascial pain (MP), disc displacement (DD), and degenerative joint disease [2].

The main goal of TMD therapy is the reduction of clinical symptoms such as pain and limited lower jaw movement [4]. Physical therapy, behavioral therapy, and the use of occlusal splints are often mentioned as successful treatment methods, with few or no differences in treatment outcomes [5]. In some studies, the combination of non-invasive modalities showed better results than the use of just one treatment option [6, 7]. The role of occlusal splints is still not fully understood, but some evidence suggests that this treatment is better than no treatment at all [8]. Treatment success is considered to be due to a combination of different factors, and one of these factors is potentially a placebo effect [9].

Research has recently suggested that oxidative stress (OS) can play a part in the pathophysiological processes that occur in TMD. OS occurs as a result of an imbalance between oxidants and antioxidants in favor of oxidants, and it is believed to play a role in various adverse processes in an organism [10]. OS is being investigated as a contributing factor to various human pathologies [11–13], including those connected to oral health [14–16].

Studies have found significant differences in the expression of OS biomarkers and antioxidants between TMD patients and healthy individuals [17, 18]. Therefore, the estimation of OS biomarkers can be used as an additional tool for early detection, diagnosis, and monitoring of TMD patients. An association between higher levels of OS markers with higher pain intensity scores has been reported [17]. Our previous study addressed the differences in oxidative status by considering pain intensity and the specific diagnostic subgroups of TMD [18]. Hence, the applied therapy might depend on the clinical subtype of TMD and can have a different effect on different individuals depending on whether they report high-intensity pain (HIP) or low-intensity pain (LIP).

The connection between OS, cortisol, and psychological stress has been previously discussed [19]. Cortisol is often mentioned as an indicator of chronic stress, because heightened cortisol release occurs during the anticipation of stress [20]. The long-lasting effects of cortisol are often connected with various diseases and conditions; oxidative damage is likewise elevated in people with diseases such as neurodegenerative, metabolic, cardiovascular conditions, or cancer [21, 22]. Although increased metabolism alone generates free radicals, glucocorticoid hormones have been shown to play both direct and indirect modulatory roles in the onset of OS [23]. Because it has been suggested that chronic stress exposure generates oxidative damage during the continuous stimulation of the hypothalamic-pituitary-adrenal axis [19], we expected that changes in oxidative status would be associated with changes in cortisol levels.

The aim of this study was to: 1) test the association between OS biomarkers, stress perception, and salivary cortisol (SC) in chronic TMD patients, and 2) to follow up on changes in OS markers and SC during 3 months of occlusal splint therapy in order to evaluate the influence of treatment on oxidative status. Our hypothesis was that there would be a positive association between SC, perceived stress, and OS biomarkers. We also hypothesized that the effects of TMD therapy on salivary oxidative status would differ between individuals with HIP and LIP as well as between MP and DD patients.

## Methods

This study was performed at the Department of Removable Prosthodontics, School of Dental Medicine, University of Zagreb with the approval of the Ethics Committee (01-PA-26-6/15, item 3.2) and regulated in accordance with the Helsinki Declaration. All subjects were informed of the procedures involved in the study and provided with written consent before being included in the research.

### Participants

A statistical power analysis was performed for sample size estimation based on data from Rodríguez de Sotillo et al. [17] which compared OS (as measured by biomarkers in saliva) in female TMD patients with high and low pain intensity. The minimum difference in salivary total antioxidant capacity (TAC) and malondialdehyde (MDA) level means was estimated to be 0.3 nmol/L and 0.49 pg/ml, respectively, and the standard deviation of TAC and MDA was expected to be 0.15 nmol/L and 0.25 pg/ml, respectively. With an alpha = .05 and power set at 80%, the projected sample size was approximately *N* = 12 (6 per group).

Participants were diagnosed according to the diagnostic criteria for TMD (DC/TMD) [2]. The inclusion criteria were a report of chronic pain lasting more than 6 months and a diagnosis of MP or DD. Only women were selected for the study because of the high prevalence of TMD in female population. The exclusion criteria were orofacial pain unrelated to TMD, degenerative joint disease, smoking, gingivitis, periodontitis, oral lesions, chronic systemic diseases (e.g., diabetes, cardiovascular diseases, cancer, and autoimmune diseases), use of supplements or medications known to affect oxidative status, and pregnancy. Patients displaying combined MP and DD diagnoses and patients who had received other TMD treatments in the preceding 6 months were also excluded. A clinician (IZA) with expertise in TMD diagnostics conducted a clinical examination of the patients.

**Fig. 1:**
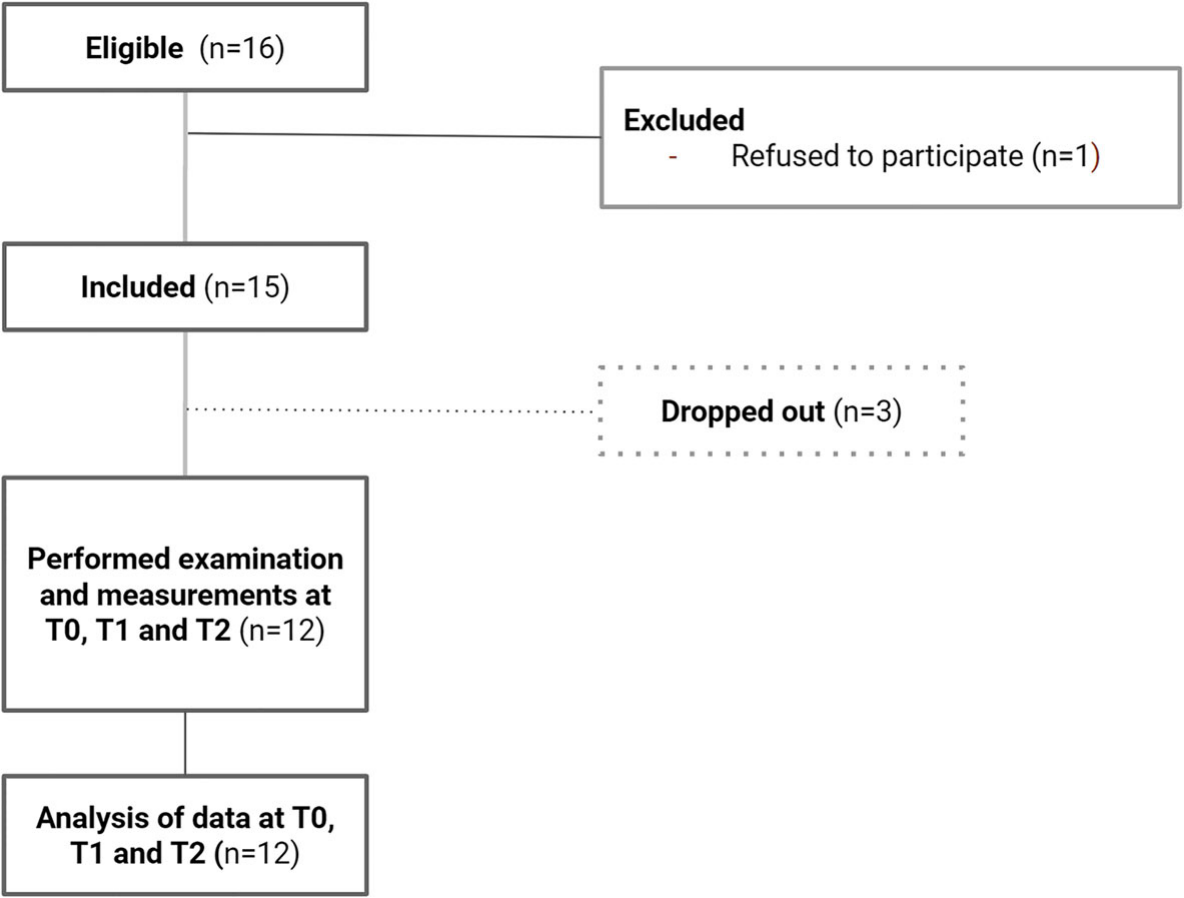
Flow diagram of the progress through the phases of the study

Sixteen female patients met the inclusion criteria. During recruitment, 1 of them refused to take part in the study due to travelling complications; thus, 15 patients were enrolled in the study, but 3 patients dropped out before the salivary measurements were performed. Ultimately, 12 patients had all of the required measurements performed at the baseline and follow-up appointments (Fig. 1).

### Grouping of patients

First, patients were grouped according to their source of pain in order to explore the interactions of OS and pain sources, and two groups were formed (6 MP and 6 DD).

Next, the patients were grouped according to pain intensity as measured by the characteristic pain intensity (CPI) using the graded chronic pain scale [2]. To calculate CPI, the means of current pain, worst pain, and average pain are computed and multiplied by 10 (CPI < 50 represents LIP and CPI ≥ 50 represents HIP). Six patients were labeled as having HIP and six as having LIP.

### Treatment protocol

All patients received a hard acrylic (Resilit-S, Erkodent, Siemensstraße 3, 72285 Pfalzgrafenweiler, Germany) occlusal splint (stabilization splint) to wear on the maxillary dental arch. The splint was made using a stone cast in an ARTEX articulator (Amann Girrbach, Austria) in the referent position of the centric relation, with a thickness of 1.5 mm at the level of the first molar. The same dental technician made all of the splints. A clinician (IZA) adjusted the splints so that opposing teeth occluded simultaneously with the splint surface. The same clinician adapted the splints as necessary at follow-up appointments.

### Outcome measures

The study followed changes in spontaneous pain as assessed by a visual analogue scale (VAS), maximal comfortable mouth opening (MCO), the Oral Health Impact Profile (OHIP-14), and the level of perceived stress (Perceived Stress Scale [PSS]).

#### VAS pain

A 100 mm horizontal VAS scale was used to evaluate spontaneous pain from the temporomandibular joint and the masticatory muscles. The range on the scale went from “no pain” (0 mm) to “worst pain imaginable” (100 mm) [24]. Only subjects with spontaneous pain of > 30 mm on the VAS were included in the study.

#### MCO

MCO was measured as the distance between the maxillary and mandibular central incisors. A pain-free opening was defined as the maximum distance the participant could open her mouth without experiencing any additional pain or discomfort [7].

#### PSS

The PSS is a standard 10-item questionnaire that assesses subjective perceptions of stress over the previous month. Response options form a 5-point Likert scale: 0 = never, 1 = almost never, 2 = sometimes, 3 = fairly often, 4 = very often. Possible scores ranged from 0 to 40, with higher numbers indicating higher levels of stress. The questionnaire was previously translated into and validated in Croatian by Hudek-Knežević et al. [25].

#### OHIP-14

The short-form OHIP-14 questionnaire [26], which has been translated into and validated in Croatian, was used to show how oral outcomes (TMD-related pain and disability) impact patients’ quality of life. Patients expressed their status through 14 questions, choosing 1 of the 5 possible answers: 0 = never, 1 = hardly ever, 2 = sometimes, 3 = fairly often, and 4 = very often. Possible scores ranged from 0 to 56. The instrument was previously validated for the evaluation of TMD patients [27].

A clinician (EV) performed a clinical examination of the patients at the baseline and follow-up appointments at the first and third months after the initiation of treatment; the clinician was blinded as to each patient’s CPI and specific diagnostic subgroup.

### Saliva collection

Five mL whole, unstimulated saliva samples were collected in graduated tubes (50 mL self-standing centrifuge tubes, Ratiolab, Germany). Saliva aliquots (1 mL) were stored at − 80 °C until the analysis. Some of the markers show significant diurnal variations; therefore, the saliva was collected in the morning (7 AM) and in the afternoon (5 PM) [16].

Morning sampling was performed at home before tooth brushing. Subjects were instructed to fast before saliva collection in the morning and to not eat or drink anything but water for at least 2 hours before sampling in the afternoon. All saliva samples were collected after rinsing the mouth with water. Brushing teeth before saliva sampling was forbidden to avoid blood contamination. Saliva was collected at follow-up measurements at the first and third months of occlusal splint therapy, following the same procedure. The sample collection and storage principles used in the study are described in detail by Alajbeg et al. [28].

### Salivary analysis of MDA, TAC, superoxide dismutase (SOD), and uric acid (UA)

The sample analysis was performed at the Department of Laboratory Diagnostics, University Hospital Center, Zagreb.

The following OS biomarkers were analyzed: MDA, TAC, SOD, and UA.

MDA was determined using the MDA Adduct Competitive ELISA kit (Kamiya Biomedical Company, Seattle, USA), which measures the MDA-stable hybrid protein adducts (MDA adducts), referred to as lipid peroxidation end products, formed by binding MDA to proteins.

TAC and SOD were measured using the commercial colorimetric reagent kits TAS and RANSOD (Randox Laboratories Ltd., Crumlin, UK) while saliva UA levels were measured using the enzymatic uricase method using commercially available reagents from Roche Diagnostics (Mannheim, Germany). The total protein concentration was determined using Roche Diagnostics’ automated turbidimetric urinary and cerebrospinal fluid total protein assay; this measuring range (0.02–2 g/L) proved to have satisfactory analytical performance for the measurement of total proteins in the saliva samples. Measurements of TAC, SOD, UA, and total proteins were performed on a Cobas c501 automated biochemistry analyzer (Roche Diagnostics, Mannheim, Germany). All OS markers’ values were normalized to the total protein concentration.

For the assays, the analytical performance (including intra- and inter-assay variability) was assessed and is available in our previously published study [28].

### Analysis of SC

Free SC was determined using a commercially available enzyme-linked immunosorbent assay (ELISA) (Demeditec Diagnostics GmbH, Germany) according to the manufacturers’ recommendations. This assay measures free SC based on the principle of competitive binding of cortisol from samples and a cortisol-enzyme conjugate to a polyclonal antibody that is pre-coated onto the microtiter wells. The resultant binding is measured spectrophotometrically and is inversely proportional to the concentration of free SC in the tested samples. The intra- and inter-assay variabilities of this assay kit are 5.8 and 6.4%, respectively, as stated by the manufacturer.

### Statistical analysis

Data analyses were performed using the Statistica 13.4.0 software package (1984–2018 TIBCO Software Inc.) with the alpha set to *p* < 0.05. Data distribution was tested using the Shapiro-Wilk test. Before performing the statistical analyses, a log transformation was used for all data that were non-normally distributed (OS markers and SC).

The Student’s t-test was used to examine differences upon admission between the TMD subgroups. The changes in the means of measured variables were analyzed using repeated measures analysis of variance (ANOVA) with time (baseline, 1st month, and 3rd months of therapy) as the within factor and pain intensity (HIP and LIP) and source of pain (MP and DD) as the between factors. Bonferroni’s post hoc test was used to show where the differences occurred. Pearson’s correlation was used to evaluate the association between changes in SC, the level of perceived stress and OS markers in the TMD subgroups.

## Results

The baseline characteristics of the patients are listed in Table 1.

**Table 1:** Pretreatment characteristics of the sample

Variable	completed (*n* = 12)		dropped out (*n* = 3)	
	mean (SD)	(95% CI)	mean (SD)	(95% CI)
Maximal comfortable mouth opening (mm)	28.00 (10.81)	21.13–34.86	31.66 (20.21)	−18.53 - 81.86
OHIP-14	28.22 (6.06)	24.47–32.18	19.66 (6.02)	4.69–34.63
PSS	16.91 (6.78)	12.60–21.22	19.33 (2.08)	14.16–24.50
VAS	70.00 (20.33)	51.51–84.83	50.00 (10.00)	25.15–74.84
CPI	51.07 (18.77)	39.14–63.01	39.33 (17.01)	− 2.29 - 81.58
PHQ-15	6.25 (3.04)	4.31–8.18	11.00 (3.61)	2.04–19.95
PHQ-9	5.00 (4.31)	2.26–7.73	8.00 (5.56)	−5.83 - 21.83
GAD-7	5.58 (5.05)	2.37–8.79	7.33 (5.85)	−7.22 - 21.88
OBC	25.16 (11.06)	18.13–32.19	33.33 (9.86)	8.82–57.84

*PSS* Perceived Stress Scale, *VAS* Visual Analogue Scale, *OHIP-14* Oral Health Impact Profile

*CPI* Characteristic pain intensity: low intensity pain < 50; high intensity pain ≥50

*PHQ-15* physical symptoms: scores of 5, 10, and 15 represent cut-points for low, medium, and high physical symptoms, respectively

*PHQ-9* depression: scores of 5, 10, 15, and 20 represent cut-points for mild, moderate, moderately severe, and severe depression

*GAD-7* anxiety: scores of 5, 10, and 15 represent cut-points for mild, moderate, and severe anxiety

*OBC* oral behaviors checklist: 0–16 -represents normal behaviors; 17–24 - occurs twice as often in TMD patients; 25–62- risk for TMD

No significant age differences in the TMD subgroups in terms of pain source (MP 38.33 ± 10.34; DD 43.00 ± 11.13; t = − 0.75, *p* = 0.46) or pain intensity (HIP 42.16 ± 13.71; LIP 39.16 ± 7.11; t = 0.47, *p* = 0.64) were found.

The mean CPI of the MP subjects (48.6 ± 13.36) and DD subjects (53.55 ± 24.12) was not statistically different (t = − 0.43, *p* = 0.66). LIP and HIP were equally distributed among the patients diagnosed with MP and with DD. Three MP subjects and 3 DD subjects had LIP (38.6 and 38.6, respectively). Similarly, 3 MP subjects and 3 DD subjects had HIP (58.53 and 68.43, respectively). However, no significant differences among the groups were found (*p* > 0.05). Therefore, patients were pooled into pain intensity subgroups, regardless of the source of pain. Similarly, patients were pooled into pain source subgroups, regardless of the pain intensity.

### Changes in OS markers observed during treatment

Table 2 lists the mean concentrations of SC as well concentrations of salivary TAC, MDA, SOD, and UA corrected to total protein concentration for all TMD patients as well as for diagnostic subgroups.

**Table 2:** Salivary OS biomarkers and cortisol concentrations in TMD patients and in specific TMD subgroups

Biomarker	time of day	TMD patients (*n* = 12)		Diagnosis (source)		Pain intensity	
				MP (*n* = 6*)	DD(*n* = 6*)	Low (*n* = 6**)	High (*n* = 6**)
		Mean	(95% CI)	Mean	Mean	Mean	Mean
TAC^**+**^ (mmol/g)	AM	4.99	2.1–7.9	5.70	4.29	2.79	7.21
	PM	4.48	2.6–6.3	4.82	4.13	3.08	5.87
MDA^**+**^ (nmol/g)	AM	370.54	130.8–610.3	466.49	274.60	222.88	518.21
	PM	761.47	368.7–1154.2	750.69	772.24	498.24	1024.69
SOD^**+**^ (U/g)	AM	4408.31	2583.4–6233.2	3976.86	4839.77	3739.95	5076.68
	PM	3388.72	1882.2–4895.2	3006.02	3771.41	3238.30	3539.13
UA^**+**^ (μmol/g)	AM	661.72	208.1–1115.3	796.86	526.58	357.73	965.71
	PM	613.07	288.9–937.18	687.96	538.19	402.90	823.25
SC (ng/ml)	AM	11.25	7.8–14.7	15.40	7.10	11.68	10.82
	PM	3.09	1.8–4.4	3.75	2.43	3.77	2.42

^**+**^Concentrations Protein corrected

*pooled regardless of pain intensity; **pooled regardless of pathology source (diagnostic subgroup)

*TAC* Total antioxidant capacity, *MDA* Malondialdehyde, *SOD* Superoxide dismutase, *UA* Uric acid, *SC* Salivary cortisol, *CI* Confidence interval, *TMD* Temporomandibular disorders, *DD* Disc displacement, *MP* Myofascial pain

The changes in the TMD subgroups’ salivary TAC, MDA, SOD, UA, and SC levels between baseline and after 1 and 3 months of treatment are presented in Table 3. Concentration is expressed in log 10 scale.

**Table 3:** Changes observed in the TMD subgroups during 3 months of occlusal split treatment

	Biomarker^a^		Diagnosis (source)				*p* ^+^ (TIME)	Pain intensity						
			MP (*n* = 6*)		DD(*n* = 6*)			Low (*n* = 6**)		High (*n* = 6**)			INTERACTION	
			Mean	(95% CI)	Mean	(95% CI)		Mean	(95% CI)	Mean	(95% CI)	p^+^ (TIME)	p^+^ (TIME*SOURCE)	p^+^ (TIME*INTENSITY)
Morning	TAC	T0	0.62	0.25–0.99	0.50	0.13–0.87	0.074	0.40	0.19–0.61	0.72	0.31–1.13	0.076	0.48	0.91
		T1	0.48	0.24–0.71	0.49	0.10–0.87		0.33	0.08–0.57	0.63	0.35–0.92			
		T2	0.37	0.04–0.70	0.34	−0.05-0.74		0.22	−0.02-0.48	0.48	0.09–0.48			
	MDA	T0	2.37	1.69–3.05	2.23	1.73–2.73	0.15	2.09	1.54–2.65	2.50	1.94–3.06	0.21	0.14	0.33
		T1	1.57	0.88–2.25	2.07	1.33–2.82		1.75	1.13–2.37	1.89	1.01–2.78			
		T2	1.81	1.27–2.34	2.28	1.75–2.81		2.07	1.61–2.53	2.01	1.31–2.71			
	SOD	T0	3.42	2.92–3.91	3.60	3.28–3.93	0.51	3.43	2.99–3.87	3.58	3.17–4.01	0.53	0.63	0.78
		T1	3.44	3.15–3.73	3.48	3.16–3.81		3.35	3.12–3.58	3.58	3.25–3.90			
		T2	3.40	3.03–3.77	3.39	3.07–3.71		3.31	3.10–3.52	3.48	3.06–3.91			
	UA	T0	2.74	2.37–3.11	2.60	2.22–2.98	0.17	2.53	2.37–2.69	2.81	2.34–3.27	0.19	0.35	0.98
		T1	2.60	2.43–2.78	2.64	2.20–3.08		2.49	2.22–2.76	2.75	2.42–3.08			
		T2	2.50	1.96–3.03	2.49	1.92–3.07		2.35	1.97–2.73	2.64	2.01–3.29			
	SC	T0	1.17	1.06–1.29	0.82	0.66–0.98	0.50	1.02	0.78–1.26	0.97	0.72–1.23	0.49	0.10	0.73
		T1	1.02	0.78–1.24	0.82	0.69–0.95		0.95	0.71–1.19	0.88	0.70–1.07			
		T2	1.05	0.84–1.26	1.00	0.66–1.33		0.99	0.72–1.26	1.06	0.77–1.34			
Afternoon	TAC	T0	0.60	0.34–0.86	0.58	0.39–0.77	0.036*	0.48	0.38–0.57	0.71	0.46–0.95	0.045*	0.17	0.50
		T1	0.50	0.26–0.74	0.47	0.10–0.84		0.43	0.22–0.63	0.54	0.17–0.92			
		T2	0.35	0.24–0.46	0.51	0.31–0.71		0.37	0.23–0.51	0.49	0.29–0.69			
	MDA	T0	2.76	2.39–3.13	2.55	1.81–3.30	0.52	2.47	1.87–3.07	2.84	2.31–3.36	0.38	0.43	0.02*
		T1	2.34	1.58–3.10	2.69	1.87–3.51		2.86	2.18–3.54	2.17	1.42–2.92			
		T2	2.31	1.63–2.98	2.53	1.82–3.24		2.62	2.49–2.75	2.21	1.28–3.14			
	SOD	T0	3.31	2.85–3.76	3.52	3.28–3.77	0.23	3.40	3.01–3.80	3.43	3.06–3.80	0.22	0.88	0.78
		T1	3.23	2.72–3.74	3.41	3.24–3.58		3.27	2.81–3.73	3.37	3.06–3.68			
		T2	3.29	3.03–3.55	3.54	3.38–3.71		3.41	3.13–3.68	3.43	3.18–3.67			
	UA	T0	2.69	2.31–3.07	2.70	2.52–2.88	0.39	2.56	2.34–2.78	2.83	2.55–3.11	0.30	0.76	0.03*
		T1	2.68	2.37–2.99	2.74	2.51–2.97		2.69	2.45–2.92	2.73	2.43–3.04			
		T2	2.56	2.19–2.93	2.67	2.33–3.01		2.51	2.23–2.77	2.73	2.34–3.12			
	SC	T0	0.50	0.19–0.80	0.28	0.04–0.60	0.99	0.50	0.20–0.80	0.28	0.04–0.60	0.99	0.24	0.04*
		T1	0.45	0.07–0.82	0.33	0.11–0.55		0.41	0.15–0.69	0.35	0.01–0.70			
		T2	1.57	0.17–0.98	0.21	0.05–0.38		0.30	0.08–0.53	0.48	0.03–0.93			

*pooled regardless of pain intensity; **pooled regardless of pathology source (diagnostic subgroup)

*TAC* Total antioxidant capacity, *MDA* Malondialdehyde, *SOD* Superoxide dismutase, *UA* Uric acid, *SC* Salivary cortisol, *CI* Confidence interval, *TMD* Temporomandibular disorders, *DD* Disc displacement, *MP* Myofascial pain, *T0* baseline, *T1* 1st month of treatment, *T2* 3rd month of treatment

^a^log transformed data; ^+^analysis of variance (ANOVA) for repeated measurements

The overall afternoon TAC did not differ between TMD subgroups (source of pain: *F* = 0.09; *p* = 0.076; pain intensity: *F* = 2.11, *p* = 0.176). but changed significantly over time (Table 3). The post hoc analysis showed that the mean afternoon TAC was significantly lower at the third month compared to the baseline (source of pain: *p* = 0.022; pain intensity: *p* = 0.033). A decrease of morning TAC was observed in all TMD subgroups. However, the change was not statistically significant.

When evaluating afternoon MDA levels, the analysis revealed a significant interaction between time and pain intensity (Wilks’s lambda = 0.42, F (2, 9) = 6.12, *p* = 0.021, η_p_^2^ = 0.33). HIP patients showed a significant reduction in afternoon MDA at the first and third month of treatment compared to the baseline (*p* = 0.012 and *p* = 0.017, respectively) while in LIP patients the levels of afternoon MDA remained unchanged. A greater reduction in afternoon MDA levels during the therapy period was observed in the MP group when compared to DD patients, but the decrease was not statistically significant.

A greater reduction in morning MDA was observed in HIP patients when compared to LIP patients, as well as in MP patients when compared to DD patients, but the difference was not statistically significant (pain intensity *F* = 0.38, *p* = 0.55; source of pain: *F* = 1.24, *p* = 0.29)).

The analysis revealed a significant interaction between time and pain intensity in terms of afternoon UA (Wilks’s lambda = 0.46, F (2, 9) = 5.11, *p* = 0.032, η_p_^2^ = 0.15). A significant reduction in afternoon UA in LIP patients was observed in the third month of treatment compared to the baseline. In HIP patients, the decrease in afternoon UA was not statistically significant.

Morning SC did not change significantly over time, but differed significantly between the MP and DD subgroups (*F* = 6.38, *p* = 0.03) with higher values in MP patients at all-time points. The effect of treatment on afternoon SC differed significantly between high and low intensity pain subgroups (Wilks’s lambda = 0.48, *F* (2, 9) = 4.71, *p* = 0.039, η_p_^2^ = 0.34). Post hoc tests revealed that afternoon SC decreased significantly in LIP patients and increased in HIP patients.

When evaluating afternoon MDA to SOD ratios (Table 4), the repeated measures ANOVA revealed a significant interaction between time and pain intensity (Wilks’s lambda = 0.40, F (2, 9) = 7.18, *p* = 0.017, η_p_^2^ = 0.30). Afternoon MDA to SOD ratios decreased significantly in HIP patients at the first and third month of treatment compared to the baseline (*p* = 0.039 and *p* = 0.034, respectively) while in LIP patients, the MDA to SOD ratios remained unchanged at the end of therapy when compared to baseline levels.

**Table 4:** Means and SD of log biomarker ratio in TMD subgroups

	Log biomarker ratio		Diagnosis (source)		Pain intensity		p (TIME*SOURCE)	p (TIME*INTENSITY)
			MP (*n* = 6*)	DD (*n* = 6*)	Low (*n* = 6**)	High (*n* = 6**)		
Morning	MDA/SOD, Mean (SD)	T0	−1.04 (0.73)	−1.37 (0.43)	−1.34 (0.80)	−1.08 (0.33)	0.12	0.56
		T1	−1.87 (0.55)	−1.41 (0.59)	−1.59 (0.68)	−1.68 (0.57)		
		T2	−1.61 (0.65)	−1.11 (0.36)	−1.24 (0.58)	−1.46 (0.57)		
Afternoon	MDA/SOD, Mean (SD)	T0	−0.54 (0.36)	−0.97 (0.74)	−0.92 (0.80)	−0.59 (0.29)	0.42	0.02
		T1	−0.89 (0.84)	−0.71 (0,78)	−0.41 (0,71)	− 1.19 (0.68)		
		T2	−0.98 (0.61)	−1.01 (0.63)	−0.78 (0.28)	−1.21 (0.76)		

*pooled regardless of pain intensity; **pooled regardless of pathology source (diagnostic subgroup)

*MDA* Malondialdehyde, *SOD* Superoxide dismutase, *DD* Disc displacement, *MP* Myofascial pain, *SD* Standard deviation, *T0* baseline, *T1* 1st month of treatment, *T2* 3rd month of treatment

### Changes in VAS, OHIP-14, PSS, and MCO

**Fig. 2:**
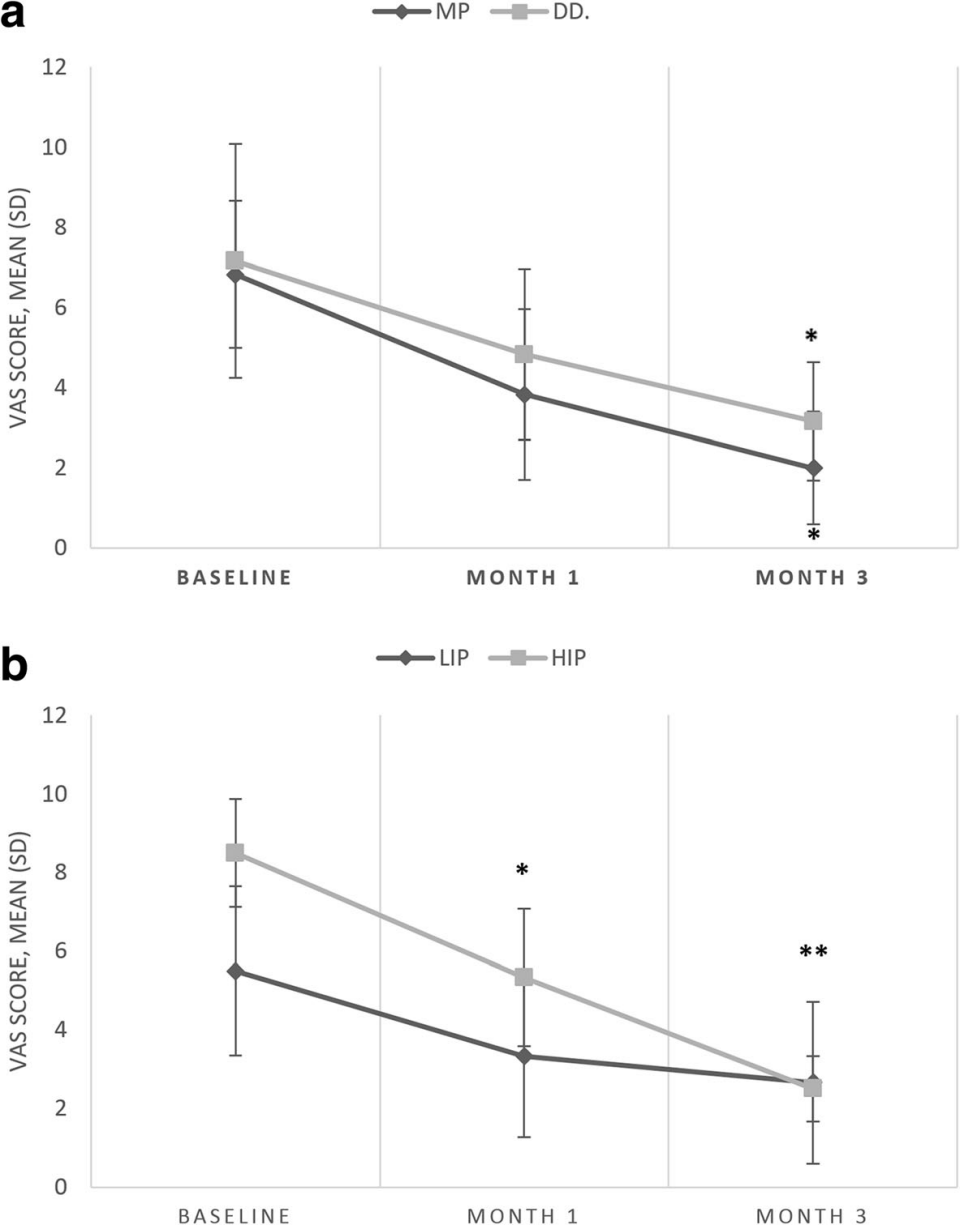
ab Visual analogue scale scores (means and standard deviations) during the 3-month interval of occlusal splint therapy. Asterisk indicates a post-hoc significant difference (within group comparisons, compared to baseline). *p < 0.05 **p < 0.001. VAS, Visual Analogue Scale; MP, myofascial pain; DD, disc displacement; HIP, high-intensity pain; LIP, low-intensity pain

When considering the source of pain, VAS scores did not differ between TMD subgroups (*F* = 1.07; *p* = 0.032), but changed significantly over time (Wilks’s lambda = 0.25, F (2, 9) = 13.5, *p* = 0.002, η_p_^2^ = 0.63) (Fig. 2). VAS scores were, however, higher in HIP patients when compared to LIP patients (*F* = 5.71, *p* = 0.038) and were significantly influenced by time (Wilks’s lambda = 0.21, F (2, 9) = 16.48, *p* = 0.001, η_p_^2^ = 0.68).

**Fig. 3:**
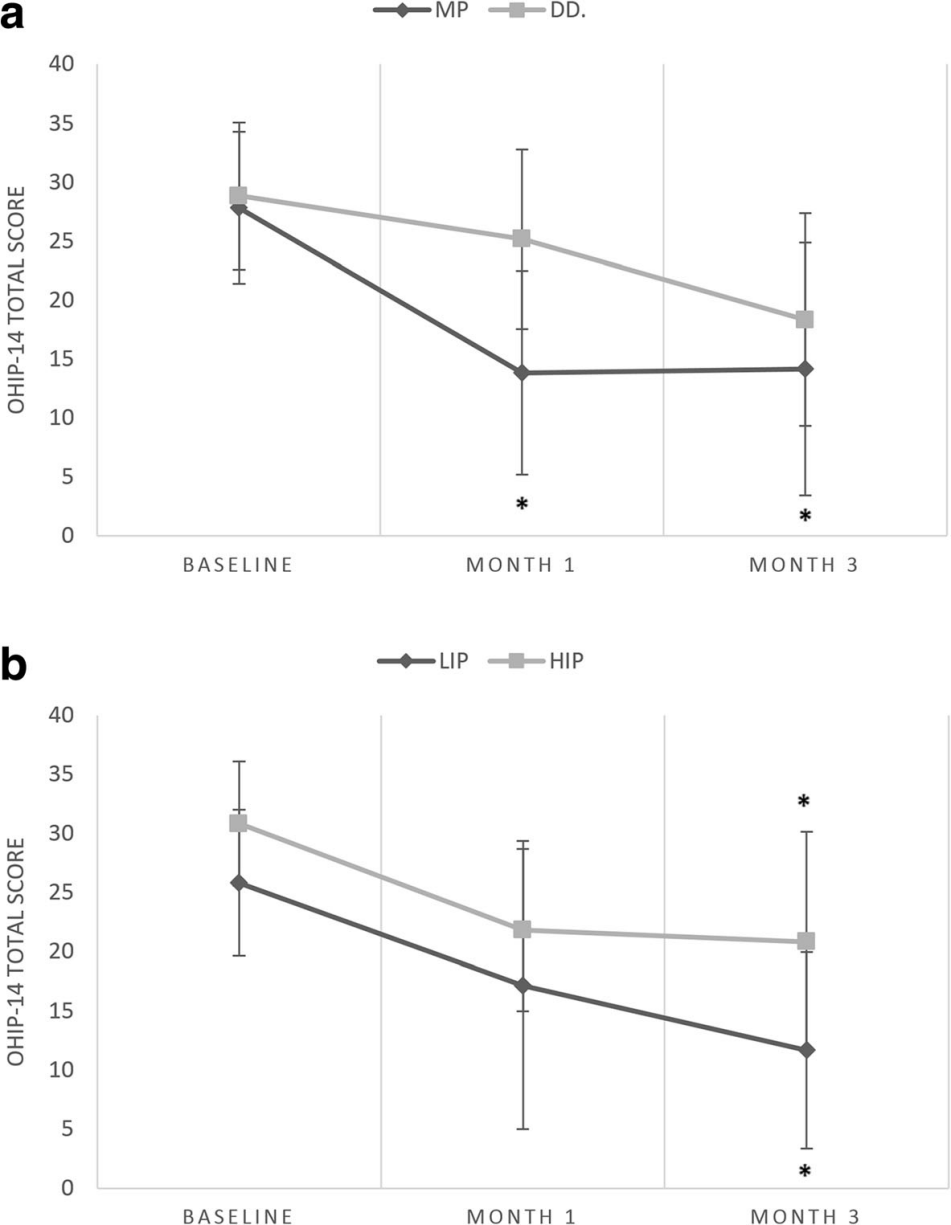
ab Oral Health Impact Profile scores (means and standard deviations) during the 3-month interval of occlusal splint therapy. Asterisk indicates a post-hoc significant difference (within group comparisons, compared to baseline). *p < 0.05 **p < 0.001. OHIP-14, Oral Health Impact Profile; MP, myofascial pain; DD, disc displacement; HIP, high-intensity pain; LIP, low-intensity pain

Oral health-related quality of life improved significantly over time in HIP and LIP patients (Wilks’s lambda = 0.37, F (2, 9) = 7.61, *p* = 0.011, η_p_^2^ = 0.53), but did not differ between groups (*F* = 2.79, *p* = 0.13). Significant improvement in OHIP-14 scores during the treatment period was observed both in MP and DD patients (Wilks’s lambda = 0.34, F (2, 9) = 8.36, *p* = 0.008, η_p_^2^ = 0.57). The effect of treatment on OHIP-14 scores was more pronounced in MP patients (Wilks’s lambda = 0.51, F (2, 9) = 4.29, *p* = 0.048, η_p_^2^ = 0.19). (Fig. 3).

**Fig. 4:**
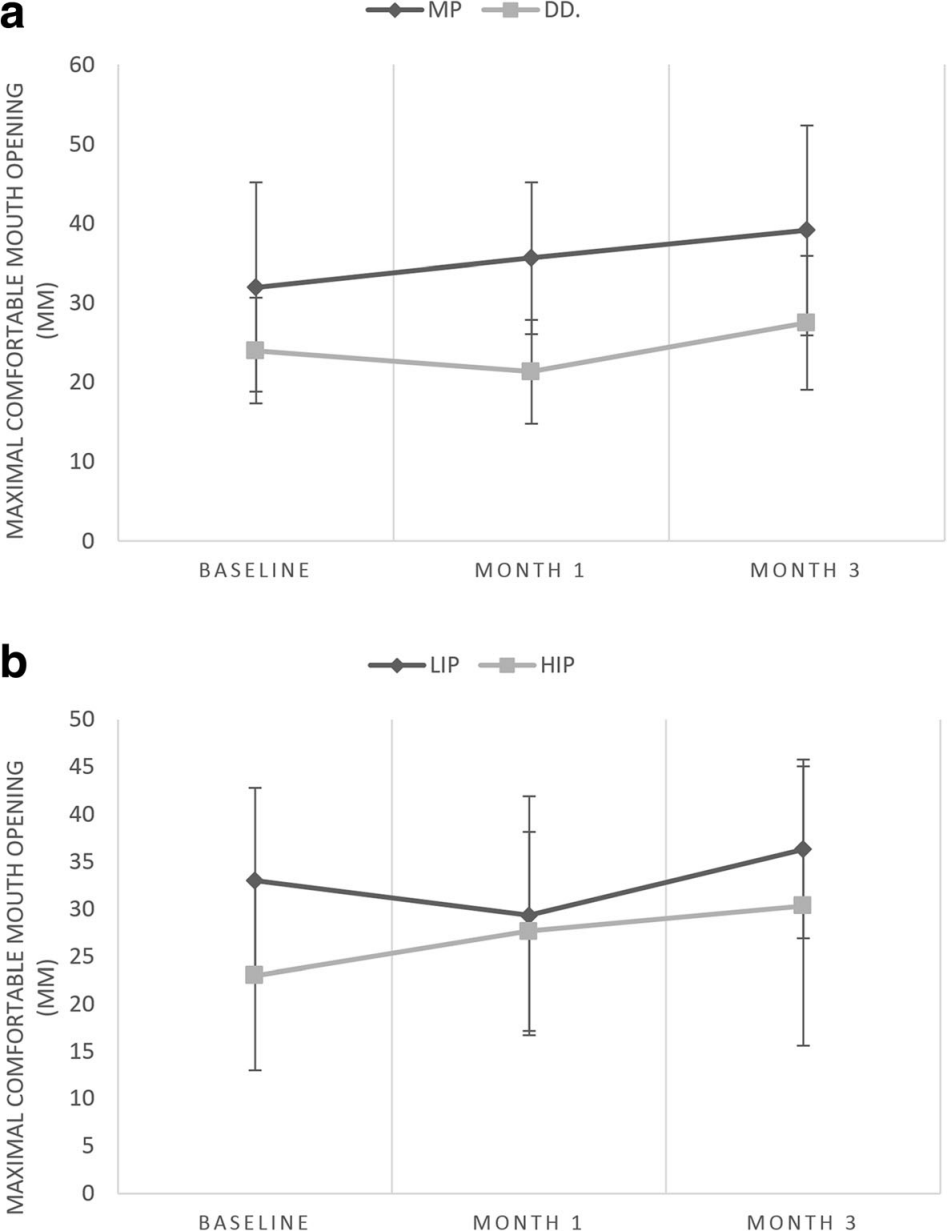
ab Maximal comfortable mouth opening (means and standard deviations) during the 3-month interval of occlusal splint therapy. MCO, maximal comfortable mouth opening; MP, myofascial pain; DD, disc displacement; HIP, high-intensity pain; LIP, low-intensity pain

Pain-free MCO increased over time and differed significantly between the MP and DD subgroups (*F* = 5.01, *p* = 0.049) (Fig. 4). At all time points, values were higher in MP patients.

**Fig. 5:**
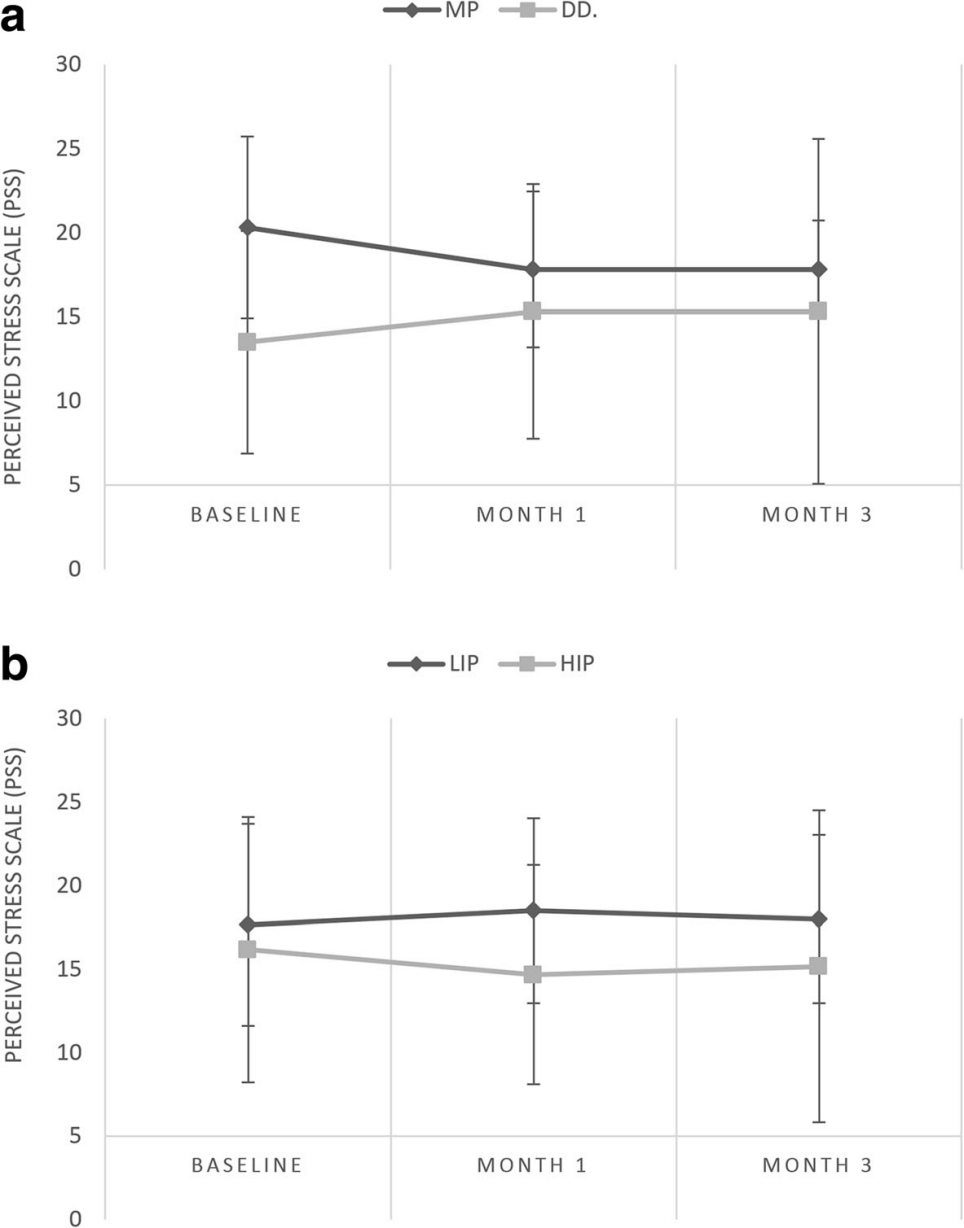
ab Change in perceived stress level (means and standard deviations) during the 3-month interval of occlusal splint therapy. PSS, Perceived Stress Scale; MP, myofascial pain; DD, disc displacement; HIP, high-intensity pain; LIP, low-intensity pain

A decrease in the levels of perceived stress was seen only in the MP group, but the changes were not statistically significant (*p* > 0.05) (Fig. 5).

### Association between PSS, SC, and OS markers

At baseline, higher levels of perceived stress were significantly associated with higher morning cortisol (*ρ* = 0.67, *p* = 0.016). However, the association between perceived stress and SC with OS markers was not significant when considering all TMD patients. When correlations were tested separately for the TMD subgroups, a significant positive correlation between morning cortisol and OS markers was found in MP patients (Table 5).

**Table 5:** Correlations among psychological stress, cortisol reactivity, and oxidative stress markers in TMD patients

				PSS	TAC	MDA	SOD	UA
TMD patients (*n* = 12)		PSS		–	,282	,076	,256	,098
		SC	morning	,673^*^	,264	,280	,086	,305
			afternoon	,216	-,252	,225	,198	,106
Diagnosis (source) pooled regardless of pain intensity	MP (*n* = 6)	PSS		–	,721*	,399	,780*	,305
		SC	morning	,795*	,781*	,064	,958*^+^	,271
			afternoon	,136	-,028	,512	,605	,226
	DD(*n* = 6)	PSS		–	-,191	-,422	,131	-,260
		SC	morning	,327	-,070	,563	,130	,231
			afternoon	,234	-,639	,017	,003	-,049
Pain intensity pooled regardless of pathology source	Low (*n* = 6)	PSS		–	,622	-,032	,521	-,276
		SC	morning	,535	,333	-,031	-,038	,267
			afternoon	-,237	-,041	,297	,219	,631
	High (*n* = 6)	PSS		–	,311	,258	,102	,285
		SC	morning	,773*	,394	,699	,255	,469
			afternoon	,731*	-,044	,543	,248	,145

*PSS* Perceived Stress Scale, *TAC* Total antioxidant capacity, *MDA* Malondialdehyde, *SOD* Superoxide dismutase, *UA* Uric acid, *SC* Salivary cortisol

^*^Note: the association was significant *p* < 0.05

^**+**^Note: the significant association was found also between morning SC and afternoon SOD (*ρ* = 0.79, *p* < 0.05)

At the end of the therapy, the percentage change in morning SC showed a significant positive correlation with reduced perceived stress (*ρ* = 0.58, *p* = 0.048) when all TMD patients were considered.

When correlations were tested separately for the TMD subgroups, a significant positive correlation between reduced perceived stress and the percentage change in morning TAC (*ρ* = 0.86, *p* = 0.025) was observed in MP patients.

## Discussion

When it comes to TAC levels in various diseases, data are inconsistent. Research addressing oxidative status in TMD patients is scarce [17, 18, 29–31], and to the best of our knowledge, no study has attempted to track changes in OS biomarker levels during TMD therapy. Rodriguez de Sotillo et al. and De Almeida and Amenábar found that TAC levels were lower in the TMD group than in control groups, indicating reduced antioxidant protection [17, 31]. However, our previous research found TAC levels to be higher in the TMD group compared to the control group. The main reason for these differences may be that in our research, we chose only female patients with chronic pain lasting 6 months or more. This six-month period provided an opportunity for the patients to adjust to potentially high oxidant levels, resulting in higher TAC levels [18]. Similar opposing results, where researchers observed higher or lower levels of TAC, have been found in other pathologies as well (e.g., atherosclerosis) [32, 33].

The higher levels of TAC previously found in TMD patients were explained as a compensatory reaction to a distorted oxidative balance [18]; therefore, the decrease in TAC levels following the drop in oxidant levels during occlusal splint therapy was expected. The decrease in TAC over time was followed by a significant reduction in the MDA levels of the HIP group. This may be due to the diminished need for antioxidative defense after the decrease in pro-oxidant activity.

Researchers have pointed out wide variability as a problem when assaying the antioxidant biomarker MDA [34]. Because of the variability in OS markers, future research will require a larger number of participants to obtain statistical significance. Nevertheless, it is important to note that MDA and the MDA to SOD ratio showed a significant decrease in the HIP group, suggesting that in this case, antioxidant biomarkers prevail over oxidants, meaning that HIP in female patients might have a stronger response to occlusal splint therapy.

UA is often mentioned as a paradoxical biomarker because it can have both antioxidant and oxidant characteristics [35]. Our study showed a significant decrease at levels similar to TAC, indicating that in this study, UA might indicate antioxidant activity. Because concentrations of salivary UA linearly follow UA levels in plasma [36, 37], and because studies suggest that UA is the antioxidant in blood [35], these results are justified.

The reduction in morning SC levels was more prominent in the MP patients when compared to the DD patients. This change in morning SC levels and the correlation between morning SC and perceived stress with OS markers observed in the MP patients implies that stress responses may have a larger impact on female patients with pain of myofascial origin. MP is often related to enhanced stress reactivity, and coping with chronic pain may lead to a larger stress response [38]. Moreover, the frequent experience of stress and anxiety may lead to the development of painful trigger points in muscles resulting in MP [39]. The minor influence of cortisol in the DD patients may be due to the fact that stress is less of a factor in disorders connected with the alteration of anatomical structures, but it surely cannot be excluded as a contributing element.

MCO improved during the treatment period, but the improvement was not statistically significant. We need to take into consideration the clinical importance of these results because an increase of only a few millimeters in MCO can have an enormous impact on a patient’s quality of life. In the current study, the MCO’s tendency to increase was a clear sign of patients’ improvement in TMD symptomatology, and it was naturally followed by an improvement in patients’ oral health-related quality of life. The significant difference observed between MP and DD patients showed that, in terms of the MCO, female MP patients had a better response to occlusal splint therapy. Research shows that there is probably no possibility of restoring the disc to its natural position, especially in chronic DD TMD [40]; hence, the altered anatomy of the joint, i.e., the biological barrier (displaced disc) that is blocking the condyle, may be the reason for a weaker MCO-related response to occlusal splint therapy.

There were a few limitations to this study. One of these was the small sample size, especially when considering the variability of the tested biomarkers. With a larger number of participants, more significant differences would most certainly occur. Second, our subjects were exclusively women; this was because few men came to the clinic with TMD-associated difficulties, which is in accordance with the fact that women are much more likely to be affected [3]. Because women are two times more likely to be affected with TMD (National Institute of Dental and Craniofacial Research) this limitation is often present in research on TMD. At the time of the current study, there are no similar studies, so there was no possibility of comparing the results. In addition, our study lacked a non-treatment or placebo group, so we cannot rule out changes that may have occurred spontaneously. Nevertheless, respondents were monitored for 3 months; thus, the results cannot be disregarded, and we strongly encourage further research on this topic.

## Conclusion

The results of this study, conducted among female, chronic TMD patients, show that during 3 months of occlusal splint therapy, the TAC levels in the patients’ saliva significantly decreased. The MDA to SOD ratios decreased, and this reduction was more pronounced in HIP patients. This decrease in the MDA to SOD ratio might indicate that the effect of splint therapy, which may be seen as a reduction of TMD symptoms in female TMD patients, might help in body’s cleansing capacity to remove free radicals. The question remains whether or not TAC decreases in this process as a result of avoiding unnecessary processes, once the increase in antioxidants effectively compensates for OS. When considering the association between SC and perceived stress with OS markers, it is important to emphasize that the positive correlation was present in female MP patients. The effect of treatment on the self-perceived quality of life was more pronounced in female MP patients while the reduction of spontaneous pain, although present in all TMD subgroups, was significantly greater in patients with HIP.

Although the study used a small sample size of only women, our results emphasize that both the source and the intensity of pain should be considered in future investigations.

## Data Availability

The data and materials generated and analyzed during the current study are not publicly available. However, all data from this study is available from the corresponding author on reasonable request.

## Abbreviations

ANOVA: Repeated-measurements analysis of variance
CPI: Characteristic pain intensity
DD: Disc displacement
ELISA: Enzyme-linked immunosorbent assay
HIP: High intensity pain
LIP: Low intensity pain
MCO: Maximal comfortable mouth opening, OHIP-14 Oral health impact profile
MDA: Malondialdehyde
MP: Myofascial pain
OHIP: Oral health impact factor
OS: Oxidative stress
PSS: Perceived stress scale
SC: Salivary cortisol
SOD: Superoxide dismutase
TAC: Total antioxidant capacity
TMD: Temporomandibular disorders
UA: Uric acid
VAS: Visual analogue scale
